# Detection of Veterinary Drugs in Food Using a Portable Mass Spectrometer Coupled with Solid-Phase Microextraction Arrow

**DOI:** 10.3390/foods13203337

**Published:** 2024-10-21

**Authors:** Hangzhen Lan, Xueying Li, Zhen Wu, Daodong Pan, Ning Gan, Luhong Wen

**Affiliations:** 1 State Key Laboratory for Managing Biotic and Chemical Threats to the Quality and Safety of Agro-Products, Zhejiang Key Laboratory of Intelligent Food Logistic and Processing, Zhejiang-Malaysia Joint Research Laboratory for Agricultural Product Processing and Nutrition, College of Food Science and Engineering, Ningbo University, Ningbo 315800, China; lixueying@nbu.edu.cn (X.L.); wuzhen@nbu.edu.cn (Z.W.); pandaodong@nbu.edu.cn (D.P.); 2 School of Material Science and Chemical Engineering, Ningbo University, Ningbo 315211, China; ganning@nbu.edu.cn; 3 China Innovation Instrument Co., Ltd., Ningbo 315000, China; wenluhong@nbu.edu.cn

**Keywords:** portable mass spectrometer, solid-phase microextraction arrow, on-site detection, mesoporous silica SBA-15, veterinary drug residues

## Abstract

A portable mass spectrometer (PMS) was combined with a mesoporous silica material (SBA-15) coated solid-phase microextraction (SPME) Arrow to develop a rapid, easy-to-operate and sensitive method for detecting five veterinary drugs—amantadine, thiabendazole, sulfamethazine, clenbuterol, and ractopamine—in milk and chicken samples. Equipped with a pulsed direct current electrospray ionization source and a hyperboloid linear ion trap, the PMS can simultaneously detect all five analytes in approximately 30 s using a one-microliter sample. Unlike traditional large-scale instruments, this method shows great potential for on-site detection with no need for chromatographic pre-separation and minimal sample preparation. The SBA-15-SPME Arrow, fabricated via electrospinning, demonstrated superior extraction efficiency compared to commercially available SPME Arrows. Optimization of the coating preparation conditions and SPME procedures was conducted to enhance the extraction efficiency of the SBA-15-SPME Arrow. The extraction and desorption processes were optimized to require only 15 and 30 min, respectively. The SBA-15-SPME Arrow–PMS method showed high precision and sensitivity, with detection limits and quantitation limits of 2.8–9.3 µg kg^−1^ and 10–28 µg kg^−1^, respectively, in milk. The LOD and LOQ ranged from 3.5 to 11.7 µg kg^−1^ and 12 to 35 µg kg^−1^, respectively, in chicken. The method sensitivity meets the requirements of domestic and international regulations. This method was successfully applied to detect the five analytes in milk and chicken samples, with recoveries ranging from 85% to 116%. This approach represents a significant advancement in food safety by facilitating rapid, in-field monitoring of veterinary drug residues.

## 1. Introduction

Veterinary drug residues are commonly found in animal-derived food products, posing significant risks to consumer health [1]. These drugs, essential for disease prevention and growth promotion in modern animal husbandry, can lead to harmful residues in food when misused. Commonly used veterinary drugs include antibacterial, antiviral, and anthelmintic agents [2]. Sulfonamides, widely used for their cost-effectiveness and broad-spectrum antimicrobial properties, pose challenges due to incomplete removal from animal products and the environment, resulting in risks such as drug resistance and urinary system damage in humans [3]. Antiviral agents were detected in chicken tissues and eggs, with long-term exposure linked to respiratory, reproductive, and nervous system impairments [4]. Anthelmintic agents, crucial for disease prevention in livestock, can cause severe toxicity in humans, affecting vital organs such as the liver, kidneys, nervous system, and bone marrow [5]. Additionally, synthetic beta-2 adrenergic stimulants, like clenbuterol and ractopamine, used to promote animal growth, can accumulate in tissues, leading to adverse effects in humans, ranging from muscle pain to life-threatening heart conditions [6]. To mitigate these risks, several regions and countries, including the European Union, the United States, and China, have implemented strict regulations to limit the use of these substances in livestock and poultry production [7,8,9]. Thus, developing rapid, accurate methods for detecting veterinary drug residues in food is critical for ensuring food safety, with increasing emphasis on on-site analytical techniques.

Traditional large-scale instrumental methods, such as thin-layer chromatography, high-performance liquid chromatography (HPLC), HPLC-mass spectrometry (HPLC-MS), gas chromatography (GC), and GC-MS, are accurate but suffer from high costs, energy consumption, and time limitations [10,11]. In contrast, a portable mass spectrometer (PMS) is a compact, field-deployable device that enables rapid, on-site detection without the need for chromatographic separation [12]. A typical PMS consists of three core subsystems: an ionization source, a mass analyzer, and a detector. The ionization source converts sample molecules into ions, which are then separated based on their mass-to-charge ratios by the mass analyzer [13,14,15]. The detector then captures and quantifies the ions, enabling the identification and quantification of multiple analytes simultaneously [16]. Commonly used ionization sources for PMS include paper spray ionization [17], ambient ionization [18], dielectric barrier discharge ionization [19], and pulsed direct current electrospray ionization (PDESI) [20]. Although PMS systems provide several advantages, such as portability, ease of use, and minimal sample preparation, they also have certain limitations, including lower resolution and sensitivity compared to benchtop instruments, which arise from their compact design and simplified vacuum systems. Nevertheless, recent advances in PDESI-based PMS configurations have improved their performance for multi-analyte detection in complex matrices, making them increasingly valuable for real-time food safety monitoring in field settings [21,22].

Several alternative techniques can reduce or eliminate the need for extensive sample preparation. These include flow injection analysis, ambient mass spectrometry, and continuous phase membrane introduction mass spectrometry [23]. However, sample pretreatment is still critical to minimize matrix interference before PMS detection, especially in complex food samples containing veterinary drugs. Common techniques include liquid–liquid extraction, dispersive liquid–liquid microextraction, dispersive solid–liquid phase extraction, stir bar sorptive extraction, and solid-phase microextraction (SPME) [24]. SPME is particularly advantageous, as it integrates sampling, separation, enrichment, and injection into a single streamlined process [25]. Conventional SPME fiber utilizes a thin fused silica or stainless-steel wire as the substrate that coated with a layer of adsorbent or absorbent. However, the SPME fiber’s small surface area limits its extraction capacity, and its mechanical fragility can reduce its lifespan, especially during repeated sampling. In contrast, the SPME Arrow—introduced in 2015—addresses these limitations by featuring a larger diameter and more robust construction, offering several key advantages over traditional SPME fibers [26]. The SPME Arrow has a 10-fold larger coating volume, which significantly enhances extraction efficiency, especially for trace-level analytes. Additionally, the increased mechanical strength of the Arrow reduces the risk of fiber breakage and improves the stability of the coating, allowing for repeated use without performance degradation [27]. These structural modifications result in higher sensitivity, improved robustness, and greater reproducibility in complex matrices. However, the efficiency of the SPME Arrow depends heavily on the adsorbent material. Mesoporous silica materials (MSM) [28] and metal–organic frameworks (MOFs) have been widely used for sample preparation [29]. MSMs offer high porosity, ordered pore channels, stability, and various functionalization options. SBA-15, SBA-16, and MCM-41 are typical MSMs with distinguished pore structures and pore sizes [27,30,31]. SBA-15 and MCM-41 exhibited two-dimensional pore structures with different pore sizes. SBA-16 exhibited a similar pore size to MCM-41 but with a three-dimensional pore structure. MOFs, with their highly porous structure composed of metal ions and organic linkers, are ideal for applications such as gas storage and separation [29]. Zirconium-based MOFs have gained wide attention because of their high physical and chemical stability and tunable pore size [32]. Representative Zirconium-based MOFs are UiO-66 and amino-UiO-66 (UiO-66-NH_2_), which have ordered two-dimensional pore structures and microporous pore sizes. Furthermore, mesoporous UiO-66 series materials are also available after a post-synthesis modification step [26]. Therefore, both MSMs and MOFs have great potential to adsorb various analytes. Therefore, a comprehensive comparison of these materials is essential for optimizing adsorption performance across a wide range of analytes.

This study presents a novel approach to detecting veterinary drugs in food products by integrating PMS with the SPME Arrow. Specifically, five veterinary drugs—amantadine, thiabendazole, sulfamethazine, clenbuterol, and ractopamine—were used as the template analytes, and milk and chicken were chosen as the template food samples. Unlike traditional methods requiring complex and expensive chromatographic separation, this method enables rapid, on-site detection with minimal sample preparation. The 30 s detection time represents a significant improvement over traditional GC and LC-based methods, which often require several min or even hours to complete. The comparison of the MSM-coated SPME Arrow, the MOFs-coated SPME Arrow, and commercially available SPME Arrows in terms of extraction capacity and selectivity further highlights the enhanced performance of the SPME Arrow. This method significantly advances on-site food safety monitoring, offering a highly sensitive and precise analytical technique capable of detecting multiple analytes efficiently. The broader contribution of this work lies in improving the rapid, in-field detection of veterinary drug residues, ensuring food safety and compliance with regulatory standards.

## 2. Materials and Methods

### 2.1. Chemicals, Standard Solutions and Instruments

N,N-dimethylformamide (DMF, 99%), polyacrylonitrile (PAN, average molecular weight: 85,000), acetonitrile (99%), amantadine (95%), ethanol (99%), ammonium hydroxide solution (NH_4_OH, 25%), tetraethyl silicate (TEOS, 95%), cetyltrimethylammonium bromide (CTAB, 99%), zirconium chloride (ZrCl_4_, 98%), terephthalic acid (H_2_BDC, 99%), 4-pentylbenzoic acid (99%), 2-amino-terephthalic acid (NH_2_-BDC, 99%), and acetic acid (99.5%) were procured from Macklin (Shanghai, China). Thiabendazole (99%) and sulfamethazine (99%) were procured from Yuanye (Shanghai, China). Clenbuterol (99%) was procured from TM Standard (Changzhou, China). Ractopamine (99%) was procured from Anpu (Shanghai, China). Pluronic P123 (average molecular weight: 5800), Pluronic F127 (average molecular weight: 12,600), SPME Arrows, and bare SPME Arrows were procured from Merck KGaA (Shanghai, China). Hydrochloric acid (HCl, 37%) was procured from Haohua Chemical Reagent Co., Ltd. (Luoyang, China). Headspace vials and PTFE/silica gel septum screw caps were supplied by Merck KGaA (Darmstadt, Germany). Ultrapure water with a resistivity of 18.25 MΩ·cm was used in this study and was produced by an ultrapure water system (Chengdu Yinghang Water Treatment Equipment Co., Ltd., Chengdu, China). Stock solutions of the five target compounds were prepared by dissolving 30 mg of each compound in 30 mL of methanol and stored at 4 °C. Working solutions were prepared by diluting the stock solutions with ultrapure water to obtain various concentrations. Scanning electron microscopy (SEM) was performed using an S-3700N instrument (Hitachi, Japan). The nanofbers were prepared by the Junada TTE-1 portable electrospinning instrument (Junada Technology Co., Ltd., Qingdao, China).

### 2.2. Synthesis of Materials

SBA-15 was synthesized by dissolving 6.4 g of Pluronic P123 in 200 mL of 2 mol L^−1^ HCl solution in a three-necked flask. The solution was stirred in a water bath at 40 °C until the P123 was completely dissolved. Next, 13.6 mL of TEOS was added dropwise while stirring at 40 °C for 20 h. The resulting white dispersion was transferred to a polytetrafluoroethylene container and placed in an autoclave reactor. Hydrothermal synthesis was conducted at 100 °C for 24 h. The solution was then filtered, and the white precipitate was collected. The precipitate was washed with deionized water at room temperature and dried overnight at 70 °C. Finally, the dried material was calcined at 550 °C for 6 h in a muffle furnace to remove the template [27].

The syntheses of SBA-16, MCM-41, and UiO-66 materials followed reported methods with slight modifications [26,27,31]. Details are provided in Section S1 of the Supplementary Material.

### 2.3. Fabrication of SPME Arrow Coatings

**Figure 1:**
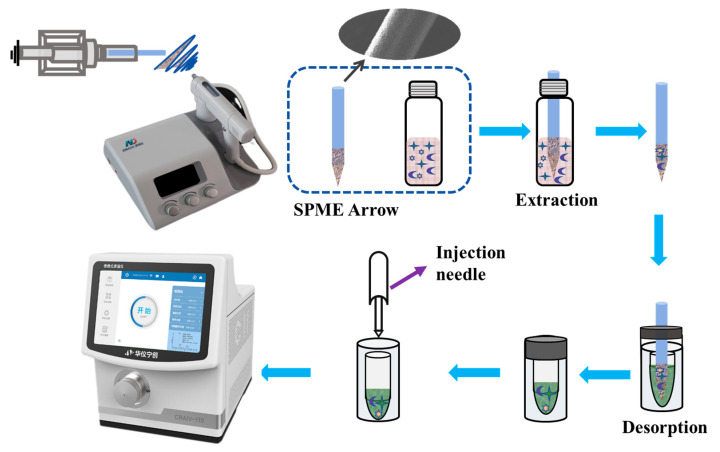
Scheme of the fabrication of the SPME Arrow and the procedures of the SPME Arrow–PMS method.

The fabrication process of the SPME Arrow is illustrated in Figure 1. First, 100 mg of PAN and 200 mg of coating materials (including mesoporous silica and MOFs) were mixed with 3 mL of DMF and stirred for 24 h. The bare SPME Arrow was cleaned by sonication with ethanol and water to remove impurities and oil residues. Next, the cleaned SPME Arrow was attached to a compact electric motor and rotated at 500 rpm during the electrospinning process. Electrospinning was conducted at 14 kV with a flow rate of 17 µL min^–1^. The distance between the syringe needle and the SPME Arrow was set at 15 cm, and the inner diameter of the spinneret was 0.6 mm. After 40 min of coating, the desired thickness was achieved. The coated area on the metal rod was 1 cm long, and any excess coating was removed with a knife. Finally, the SPME Arrow was immersed in an ethanol solution to facilitate the solvent exchange reaction and then dried in an oven at 80 °C for 12 h.

### 2.4. Portable Mass Spectrometer Analysis

Mass spectrometry analysis was conducted using a PMS “CRAIV-110” manufactured by Ningbo Hua Yi Ning Chuang Intelligent Technology Co. (Ningbo, China) [21]. The PMS was equipped with a PDESI source and a hyperbolic ion trap. The inlet temperature was maintained at 200 °C, while the PDESI voltage was set to 2 kV. The molecular pump speed was 1375 Hz. The sheath and auxiliary gas flow rates were both set to 2.5 L min^−1^. Ultra-pure helium (≥99.999%) was used as the carrier gas. The PMS resolution, determined using the full-width at half-maximum method, ranged from 0.5 to 1.2 Da, depending on the *m*/*z* values of the compounds. The selected ion monitoring mode was used for quantitative detection of the analytes. The PMS was configured to allow for the simultaneous detection of five veterinary drugs (sulfamethazine, thiabendazole, amantadine, ractopamine, and clenbuterol) within 30 s. This short detection time significantly increases throughput, making the method highly suitable for large-scale screening and real-time monitoring in food safety applications. The *m*/*z* transitions of the five target analytes and two competing compounds (bisacodyl and metronidazole) are shown in Table 1. The portability and user-friendly interface of the PMS make it highly suitable for field applications, allowing rapid, on-site detection of veterinary drug residues. The ability to perform real-time analysis without chromatographic separation enhances its practicality for food safety monitoring.

### 2.5. Milk and Chicken Samples Preparation

Milk and chicken samples were purchased from a supermarket in Ningbo, China. Each sample was prepared in triplicate (n = 3) and each sample was measured three times using the PMS. For milk sample preparation, 1 g of zinc sulfate and 1 g of K_4_[Fe(CN)_6_]-3H_2_O were added to 100 mL of milk. The mixture was vortexed for 20 s and centrifuged at 10,000 rpm for 3 min to remove the precipitate. The supernatant was filtered through a 0.22 μm membrane and stored at 4 °C. For chicken sample preparation, the meat was blended using a high-speed crusher and stored at −18 °C. Next, 5.0 g of chicken was homogenized with 20 mL of acetonitrile in a 50 mL centrifuge tube for 2 min. The mixture was centrifuged at 4500 rpm for 5 min, and the supernatant was filtered through a 0.22 μm filter.

### 2.6. SPME Arrow Procedure

Figure 1 shows the SPME Arrow sampling procedures. First, 10 mL of the working solution or food sample was added to a 10 mL sample vial. The SPME Arrow was then immersed in the solution in direct immersion mode to enrich the analytes. The extraction temperature was maintained at 25 °C, and the agitation speed was set to 750 rpm. Static desorption was performed by exposing the SPME Arrow to 200 μL of acetonitrile at room temperature. The desorbed analytes were then analyzed using the PMS. After each extraction and desorption cycle, the SPME Arrow was washed with 200 μL of acetonitrile for 20 min to prevent residual analytes from affecting subsequent experiments. Each SPME Arrow sampling and analysis was performed in triplicate to ensure repeatability and statistical robustness.

## 3. Results

### 3.1. Feasibility Testing of the PMS

The PMS offers a rapid and efficient solution for the simultaneous detection of five veterinary drugs, significantly reducing the typically time-consuming process of residue detection in food samples [21,22,33,34]. Without the need for chromatographic separation, the PMS demonstrates excellent potential for on-site applications, making it an invaluable tool for real-time food safety monitoring.

**Figure 2:**
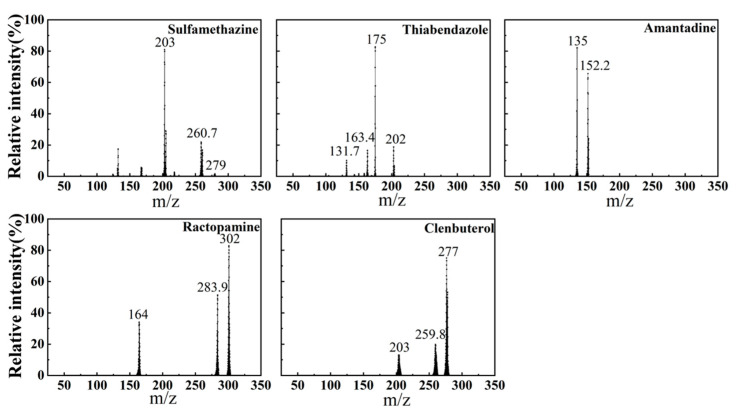
Representative mass spectra of sulfadimethoxine, thiabendazole, amantadine, ractopamine, and clenbuterol.

To confirm the feasibility of the PMS for detecting multiple veterinary drugs simultaneously, a standard mixture (100 µg mL^−1^) containing the five target analytes (sulfamethazine, thiabendazole, amantadine, clenbuterol, and ractopamine) was analyzed. Additionally, individual analyses of each compound were performed to establish baseline spectra for comparison. This allowed us to ensure that co-elution or ion suppression did not occur when the compounds were tested as a mixture. The mass spectra of the single analytes were consistent with those observed in the mixture, with no significant shifts in *m*/*z* values or changes in fragmentation patterns, confirming that the presence of multiple analytes did not interfere with their detection. These results validate the compatibility of the PMS system for the simultaneous detection of the five target analytes (Figure 2), providing a reliable foundation for subsequent method validation.

Specifically, sulfadimethoxine exhibited a quasi-molecular ion peak at *m*/*z* 279 in positive ion mode, with secondary fragment ions at *m*/*z* 260.7 and *m*/*z* 203. Thiabendazole displayed a quasi-molecular ion peak at *m*/*z* 202, along with secondary fragment ions at *m*/*z* 131.7, *m*/*z* 163.4, and *m*/*z* 175. Amantadine’s quasi-molecular ion peak was identified at *m*/*z* 152.2, with secondary fragment ions at *m*/*z* 135. Ractopamine exhibited an excimer ion peak at *m*/*z* 302, with secondary fragment ions at *m*/*z* 283.9 and *m*/*z* 164. Clenbuterol’s quasi-molecular ion peak was observed at *m*/*z* 277, with fragment ions at *m*/*z* 203 and *m*/*z* 259.8. The preliminary feasibility tests demonstrated that the PMS could simultaneously detect the five veterinary drugs (sulfamethazine, thiabendazole, amantadine, clenbuterol, and ractopamine) with distinct and stable spectra for each analyte. This confirms the system’s capability to handle multi-analyte detection. These initial results establish a strong basis for the subsequent validation and optimization of the method. Additionally, the compact design and low weight of the PMS system (<30 kg) support its potential for on-site applications.

### 3.2. Comparison of the Extraction Performance of Seven Porous Nanomaterials

**Figure 3:**
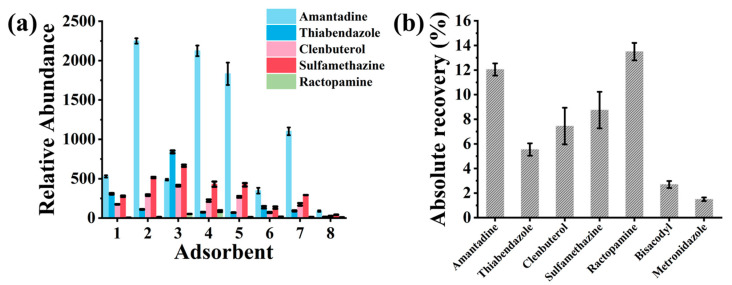
(**a**) Comparison of coating materials: (1) UiO-66-NH2-50%, (2) MCM-41, (3) 4-meso-UiO-66, (4) SBA-15, (5) SBA-16, (6) HCl-meso-UiO-66, (7) meso-UiO-66, and (8) PAN; (**b**) absolute recoveries of five veterinary drugs and two competing compounds by SBA-15-SPME Arrow. The mixed solution contains 3 μg mL^−1^ of amantadine, thiabendazole, sulfamethazine, clenbuterol, ractopamine, and 300 μg mL^−1^ of bisacodyl and metronidazole.

The extraction efficiency of seven porous nanomaterials—SBA-15, SBA-16, MCM-41, UiO-66-NH_2_-50%, Meso-UiO-66, HCl-Meso-UiO-66, and 4-Meso-UiO-66—coated on SPME Arrows (with PAN as the adhesive agent) was evaluated for extracting five veterinary drugs in the mixed standard solution (3 µg mL^−1^) [26,27,31]. Among these materials, SBA-15 exhibited the highest adsorption capacity and superior selectivity for all analytes (Figure 3a). This performance can be attributed to SBA-15′s large surface area (~720 m^2^ g^−1^), well-ordered mesoporous structure, and large pore volume (~0.94 cm^3^ g^−1^), which provide numerous active sites for adsorption and facilitate efficient diffusion of analytes into the pores [27]. Additionally, the silica surface of SBA-15 promotes hydrogen bonding and van der Waals interactions with the functional groups of the veterinary drugs, further enhancing selective adsorption [35]. Compared to MCM-41 and UiO-66, SBA-15′s superior adsorption performance is likely due to its optimal pore size and stability, allowing for repeated use without performance loss [27]. SBA-15′s enhanced extraction capacity offers a significant advantage for detecting trace amounts of veterinary drugs in complex matrices. By improving the sensitivity and selectivity of the SPME Arrow, SBA-15 enhances the accuracy and reliability of on-site detection methods, making it a valuable tool for food safety monitoring. These results highlight SBA-15′s potential for broader applications in analytical chemistry.

Further selectivity testing of the SBA-15-SPME Arrow in the presence of two additional antibiotics, bisacodyl, and metronidazole, showed no significant impact on the adsorption of the five target veterinary drugs, despite the higher concentrations of these competing compounds (Figure 3b). These findings demonstrate the superior extraction capacity and selectivity of SBA-15 coatings, even in the presence of potential interferences. As a result, SBA-15 was selected for use in subsequent experiments.

### 3.3. Evaluation of the Electrospinning Conditions of SBA-15 Coating

The effect of electrospinning slurry composition on the extraction efficiency of the SBA-15-SPME Arrow for the five target analytes was investigated (Figure S1a). The amount of SBA-15 in the SPME Arrow coating was varied to assess its impact on adsorption capacity. Increasing the SBA-15 content from 100 mg to 300 mg enhanced adsorption efficiency due to the increased surface area available for analyte interaction. However, beyond 200 mg, the improvement became marginal, likely because the additional SBA-15 did not significantly increase the effective surface area exposed to the sample. Optimal adsorption performance was achieved when 100 mg of PAN and 200 mg of SBA-15 were mixed with 3 mL of DMF (Figure S1a). This composition appears to strike the best balance between the mechanical properties of the coating and its adsorption capacity. PAN, as the polymeric support, provides flexibility and durability, ensuring good adhesion to the SPME Arrow while withstanding multiple extraction cycles [26,27]. The higher proportion of SBA-15 increases surface area and adsorption capacity, enabling more efficient extraction of veterinary drugs. Preliminary tests confirmed that this combination provides the best performance in terms of coating stability and extraction efficiency.

**Figure 4:**
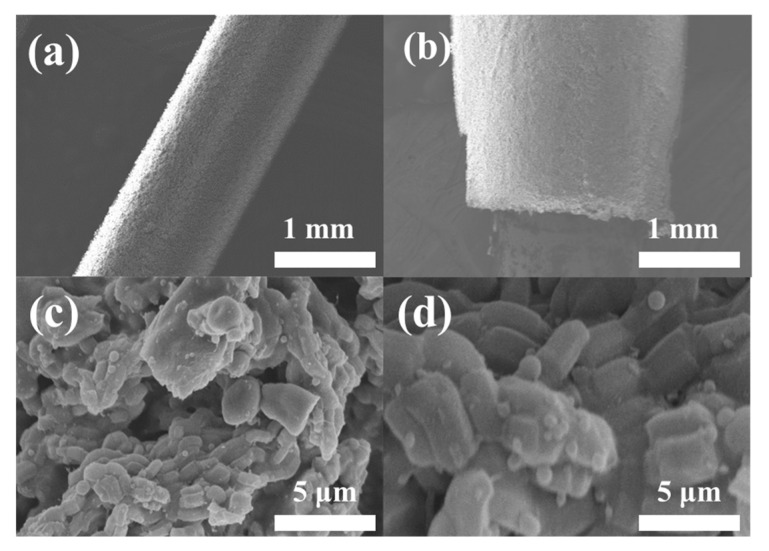
SEM images of SBA-15 coating (40 min electrospinning) with different magnifications: (**a**) 30×, (**b**) 50×, (**c**) 10,000×, and (**d**) 30,000×.

The thickness of the SBA-15 coating increased from 135 µm to 300 µm as the electrospinning time was extended from 20 to 60 min (Figure S2). Correspondingly, the extraction capacity of the SBA-15 coating for the five veterinary drugs significantly improved (Figure S1b). However, extending the electrospinning time beyond 40 min did not notably enhance extraction capacity, likely due to the excessive thickness of the coating, which could impede diffusion pathways and slow adsorption kinetics [25]. Therefore, a 40 min electrospinning duration was selected for fabricating the SBA-15-SPME Arrow (Figure S1b). SEM images of the SBA-15-SPME Arrow (40 min electrospinning), captured at different magnifications, confirmed the uniformity of the SBA-15 coating on the bare SPME Arrow (Figure 4).

### 3.4. Evaluation of SPME Procedure Parameters

Optimizing the SPME procedure is essential to maximize extraction efficiency. Critical extraction and desorption parameters were investigated to determine the optimal conditions.

#### 3.4.1. Evaluation of Sample Solution

**Figure 5:**
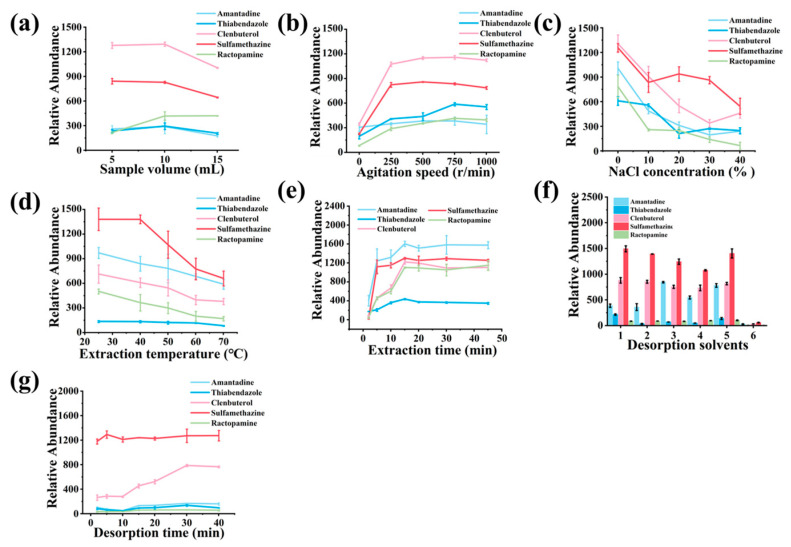
Optimization of SPME Arrow procedures: (**a**) sample volume, (**b**) NaCl concentration, (**c**) agitation speed, (**d**) extraction temperature, (**e**) extraction time, and (**f**) desorption solvent ((1) acetonitrile, (2) acetone, (3) methanol, (4) acetonitrile/water = 9:1 (*v*/*v*), (5) acetonitrile/water = 7:3 (*v*/*v*), (6) acetonitrile/water = 1:9 (*v*/*v*)), and (**g**) desorption time.

The effect of sample volume on the extraction efficiency of the SBA-15 coating was first examined. Although smaller sample volumes theoretically enhance extraction efficiency due to stronger turbulence effects and faster analyte diffusion [25], comparable extraction efficiencies were observed with sample volumes of 5 mL and 10 mL (Figure 5a). In order to avoid the analytes vaporizing in the headspace of the sample vial and the mechanical destruction to the SPME Arrow coating by the magnetic stirrer during the agitation, a sample volume of 10 mL was selected for subsequent experiments.

The extraction efficiency of the SBA-15 coating for all analytes decreased as the NaCl concentration increased from 0% to 40% (Figure 5b). This decline is attributed to the increased solubility of the analytes in water at higher salt concentrations, which reduces the distribution constant between the coating and the aqueous phase [36]. Therefore, no salt was added to the extraction solution in subsequent experiments.

#### 3.4.2. Evaluation of Extraction Parameters

Agitation speed directly impacts the mass transfer of analytes from the sample matrix to the adsorbent. Speeds ranging from 0 to 1000 rpm were tested, and increasing the agitation up to 750 rpm improved extraction efficiency (Figure 5c). This effect is due to the reduction in the boundary layer around the SPME Arrow, promoting faster mass transfer of analytes to the SBA-15 surface [25,26,27]. However, speeds above 750 rpm led to vortex formation, reducing the contact time between the adsorbent and the sample, thus decreasing extraction efficiency. Therefore, 750 rpm was chosen as the optimal stirring rate, providing sufficient turbulence for analyte transfer while maintaining good contact between the coating and the sample solution.

Extraction temperature plays a crucial role in adsorption by influencing analyte diffusion and interaction with the adsorbent. Temperatures ranging from 25 °C to 70 °C were tested, with optimal extraction efficiency observed at 25 °C (Figure 5d). Lower temperatures reduced analyte diffusion rates into the SBA-15 coating, resulting in low adsorption efficiency, while temperatures above 30 °C caused desorption of the analytes from the SPME Arrow due to increased molecular motion, disrupting adsorption equilibrium. Thus, 25 °C was selected as the optimal temperature to balance adsorption efficiency, ensuring efficient extraction while minimizing evaporation.

Extraction time was varied between 2 and 45 min to determine the optimal duration for maximum analyte recovery. Extraction efficiency increased with time as more analytes diffused into the SBA-15 pores during prolonged contact. However, beyond 15 min, extraction efficiency was saturated, indicating that equilibrium had been reached, and no further adsorption occurred (Figure 5e). Longer extraction times did not improve efficiency and could result in analyte desorption back into the solution. Thus, 15 min was selected as the optimal extraction time, balancing maximum efficiency with practical analysis time. Compared to the reported SPME coatings, the SBA-15/PAN coating showed not only a wider cover range for the five veterinary drugs but also the shortest equilibrium time [37,38,39,40], which is beneficial for on-site applications.

#### 3.4.3. Evaluation of Desorption Parameters

The desorption time and solvent were optimized to ensure complete recovery of analytes from the SPME Arrow after extraction. Various desorption times (from 1 to 40 min) and solvents (acetonitrile, acetone, methanol, acetonitrile/water = 9/1 (*v*/*v*), acetonitrile/water = 7/3 (*v*/*v*), and acetonitrile/water = 1/9 (*v*/*v*)) were tested. Among the solvents, acetonitrile exhibited the highest desorption efficiency due to its lower polarity compared to methanol and its stronger elution efficiency as a polar protonic solvent (Figure 5f) [41]. As a result, acetonitrile was selected as the optimal desorption solvent. Subsequent optimization of desorption time revealed that 30 min was sufficient to achieve maximum desorption of the target analytes (Figure 5g). This desorption time is relatively long because static-state desorption mode was utilized, but it is still faster than the reported method, which needs 35 min even when agitated desorption mode is utilized [37]. In general, the shortcoming of the long desorption time of static-state desorption can be compensated by a higher enrichment factor and coating lifetime because much less desorption solvent is used, and less mechanical destruction occurs during the agitation.

### 3.5. Extraction Performance of the SBA-15-SPME Arrow

**Figure 6:**
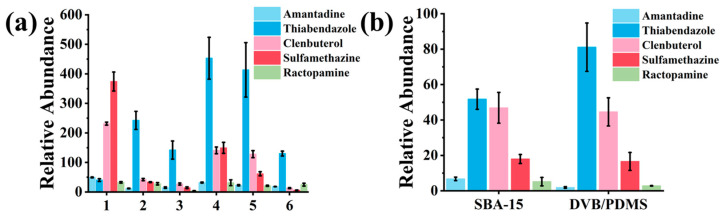
(**a**) Comparison of extraction efficiency between SBA-15-SPME Arrow and commercial SPME Arrow under optimal conditions for detecting veterinary drugs: (1) SBA-15-SPME Arrow, (2) DVB/Carbon WR/PDMS SPME Arrow, (3) polyacrylate SPME Arrow, (4) DVB/PDMS SPME Arrow, (5) Carbon WR/PDMS SPME Arrow, and (6) PDMS SPME Arrow; (**b**) comparison of SBA-15 and DVB/PDMS SPME Arrow in milk.

The extraction efficiency of the SBA-15-SPME Arrow was compared with commercially available SPME Arrows with different coatings (Carbon WR/PDMS, DVB/PDMS, PDMS, DVB/Carbon WR/PDMS, and Polyacrylate) under their optimal extraction and desorption conditions (Figures S3–S7) in a mixed standard solution (50 ng mL^−1^). As shown in Figure 6a, the DVB/PDMS SPME Arrow demonstrated the highest extraction capacity among the five commercial SPME Arrows. However, the SBA-15-SPME Arrow exhibited significantly higher extraction efficiency for all analytes except thiabendazole. Additionally, the extraction performance of the SBA-15-SPME Arrow and the DVB/PDMS SPME Arrow was compared for the five analytes in milk samples. As shown in Figure 6b, the SBA-15-SPME Arrow exhibited much higher extraction capacity and selectivity for amantadine, clenbuterol, sulfamethazine, and ractopamine, while the DVB/PDMS SPME Arrow showed slightly higher extraction efficiency for thiabendazole in milk samples. Overall, the SBA-15-SPME Arrow demonstrated excellent extraction ability and selectivity for the five veterinary drugs compared to the commercially available SPME Arrows, confirming its superior extraction efficiency. This enhanced performance is crucial for improving the precision of on-site analytical methods, especially in complex food matrices where interference from other compounds can be problematic. The ability of SBA-15 to provide a higher extraction capacity and greater selectivity improves the accuracy of quantitative analyses, enabling faster and more reliable results in the field. These findings highlight the potential for broader adoption of SBA-15-SPME Arrows in regulatory food safety monitoring, where rapid and accurate detection of contaminants is essential.

### 3.6. Reusability, Reproducibility, and Carryover Effect of the SBA-15-SPME Arrow

**Figure 7:**
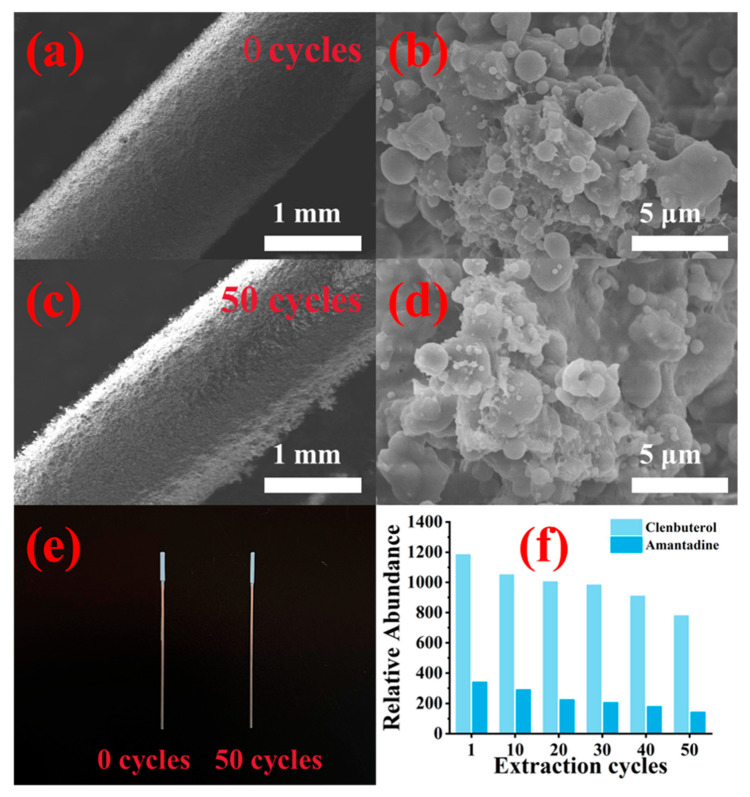
SEM images of (**a**,**b**) the new SBA-15-SPME Arrow and (**c**,**d**) the SBA-15-SPME Arrow used for 50 cycles; (**e**) macro-photos of the new SBA-15-SPME Arrow and the SBA-15-SPME Arrow used for 50 cycles; (**f**) reusability of SBA-15-SPME Arrow.

The SEM images reveal a highly porous and uniform SBA-15 coating (Figure 7), which enhances surface interaction with analytes. This morphology contributes to increased adsorption efficiency, especially in complex matrices like food samples. SEM images (Figure 7a,b) illustrate that the SBA-15-SPME Arrow maintained structural integrity even after 50 extraction and desorption cycles. This robustness is critical for applications where repeat use is necessary, such as in large-scale food safety monitoring programs. The SPME Arrow maintained a uniform SBA-15 coating on the metal rods, with minimal changes in appearance or internal structure, even after extended use. Figure 7c,e further confirmed these observations, showing no discernible changes in the coating after 50 adsorption/desorption cycles. Figure 7f presents the trend in extraction efficiency for clenbuterol and amantadine over 50 cycles, showing a slight decline in efficiency by the 50th cycle. However, the SPME Arrow remained effective in extracting the target analytes, demonstrating commendable reusability.

Further reproducibility tests, including intra- and inter-batch reproducibility, were conducted. The relative standard deviation (RSD) within the same batch remained below 7.0%, indicating consistent performance. Similarly, the RSD across different batches was below 9.3%, demonstrating robust reproducibility across batches. Quantification of residual rates was performed by comparing the mass of target compounds in three desorptions against the initial 30 min desorption (Figure S8). The results suggest that 200 µL of acetonitrile for 30 min was sufficient to desorb most analytes from the SBA-15-SPME Arrow.

The reusability, reproducibility, and carryover test of the SBA-15-SPME Arrow were initially evaluated using standard mixtures to establish a controlled baseline for its mechanical robustness and coating stability. Subsequent quantitative analysis with real samples employed matrix-matched calibration to minimize potential matrix effects and improve quantification accuracy. This ensures that, even in complex matrices, the analytical performance of the SPME Arrow remains reliable. A more comprehensive evaluation of complex food matrices will be explored in future work.

### 3.7. Method Validation

Matrix effects are common in complicated matrix samples and can negatively impact the accuracy and precision of a method. Matrix effects occur when co-extracted matrix components interfere with ionization, leading to signal suppression or enhancement, which can cause inaccurate quantification [42]. Matrix effects were assessed by comparing the calibration curve slopes of real samples (K_1_) to those of standard solutions (K_2_), using the equation Matrix effects = (1 − K_1_/K_2_) × 100%. Matrix effects values ranging from −20% to 20% indicate slight signal suppression or enhancement, while values between −50% and 50% indicate moderate effects and values below −50% or greater than 50% indicate strong effects [26]. The matrix effects for the five target analytes ranged from 51.3% to 69.9%, indicating severe matrix effects in the milk and chicken samples. Therefore, matrix-matched calibration curves were employed to compensate for these matrix effects, which are common in complex food samples. By preparing calibration standards in the same matrix as the samples, this method accounts for matrix-induced variations in analyte response, ensuring accurate and reliable quantification [43]. This approach improves precision and accuracy, maintaining consistent sensitivity across batches and preventing over- or underestimation of analyte concentrations.

Although using an internal standard is a widely recommended approach to further enhance precision and accuracy, it was not incorporated in this study because selecting a single internal standard that can effectively compensate for the matrix effects and extraction efficiency of all five target analytes poses a challenge, as each compound has unique chemical properties. Instead, matrix-matched calibration was chosen as a more reliable and straightforward approach, effectively correcting for matrix-related biases and ensuring robust quantification. As shown in Table 2, the intra-day and inter-day RSDs were within acceptable ranges, demonstrating that the method achieves high reproducibility and precision without an internal standard. Future work will aim to investigate and incorporate appropriate internal standards for each class of veterinary drugs to further enhance the method’s analytical performance.

The linear range, correlation coefficient (R), limit of quantification (LOQ), limit of detection (LOD), and reproducibility of the SBA-15-SPME Arrow–PMS method obtained using the matrix-matched calibration strategy were evaluated, as shown in Table 2. The correlation coefficients (R) for all five targets were greater than 0.995. The LOD and LOQ, calculated based on signal-to-noise ratios of 3 and 10, respectively, ranged from 2.8 to 9.3 µg kg^−1^ and from 10 to 28 µg kg^−1^, respectively, in milk. The LOD and LOQ ranged from 3.5 to 11.7 µg kg^−1^ and from 12 to 35 µg kg^−1^, respectively, in chicken. All these results meet the requirements of the regulations of the European Union, the United States, and China (Table 3) [7,8,9]. Intraday and interday RSDs ranged from 3.2% to 10.3% and from 8.5% to 13.4%, respectively, indicating good precision.

A comparative study showed that the SPME Arrow–PMS method exhibited a much shorter detection time and a comparable sensitivity, linear range, and recovery compared to other HPLC-based methods (Table 4) [44,45,46,47]. Although LC-MS methods can detect much lower levels of veterinary residues due to their advanced instrumental design, they require 10–15 min per sample, even with UPLC systems. Their detection time is significantly longer than that of the SPME Arrow–PMS method, which completes the analysis of five veterinary drugs in 30 s. Additionally, LC-MS methods are less suited for on-site food safety monitoring compared to this portable, rapid method.

### 3.8. Real Sample Analysis

The applicability of the SPME Arrow–PMS method was evaluated using milk and chicken samples. Since no target analytes were detected in these samples, spiking was performed at three concentration levels. According to Chinese National Standard GB 31650, the maximum residual limits for sulfadimidine and thiabendazole in meat and milk are 25 µg kg^−1^ and 100 µg kg^−1^, respectively. Additionally, amantadine, clenbuterol, and ractopamine are prohibited in animal-derived food products. Given the LOQ for thiabendazole (25–28 µg kg^−1^, Table 2), spiking levels for amantadine, clenbuterol, sulfadimidine, and ractopamine were set at 25, 100, and 200 µg kg^−1^, while spiking levels for thiabendazole were set at 30, 100, and 300 µg kg^−1^. As shown in Table 3, recoveries ranged from 87% to 107% for milk samples and from 85% to 116% for chicken samples, with RSDs between 2.1% and 14.7%. The developed SPME Arrow–PMS method exhibited excellent accuracy and precision for the quantitative and qualitative determination of the five analytes in complex food matrices.

Despite the acceptable accuracy and precision shown in the spiking experiments, the current method’s sensitivity for detecting amantadine, clenbuterol, and ractopamine at trace levels remains limited. This could be a reason why no positive residues were found in the unspiked milk and chicken samples. Further optimization may be required to meet the stricter regulatory limits in certain regions.

## 4. Conclusions

In this study, a simple, rapid, and on-site applicable SPME Arrow–PMS method for the selective and quantitative detection of five veterinary drugs in food was developed. This method eliminates the need for chromatographic separation, allowing detection within 30 s with LODs ranging from 2.8 to 9.3 µg L^−1^. The durability and enhanced extraction efficiency of the SBA-15-SPME Arrow make it a strong candidate for incorporation into routine food safety inspections and other high-throughput screening environments. Compared to commercially available SPME Arrows, the SBA-15-coated Arrow showed superior extraction performance, offering greater sensitivity and selectivity while requiring only 1 µL of the desorbed sample. These features make it highly suitable for real-time food safety monitoring. The SBA-15-SPME Arrow not only improves sensitivity and precision but also reduces costs, making it a viable tool for large-scale screening of veterinary drug residues in food products. The SPME Arrow–PMS method offers a portable, cost-effective solution for on-site food safety monitoring, enhancing both detection speed and accuracy. Its potential for routine screening of veterinary drug residues in dairy products and meat suggests broader utility in routine food safety inspections.

## Figures and Tables

**Table 1:** The chemical structure and transition (*m*/*z*) of sulfamethazine, thiabendazole, amantadine, ractopamine, clenbuterol, bisacodyl, and metronidazole.

Compounds	Structure	Transition (*m*/*z*)	Qua ^a^ Quant ^b^
Sulfamethazine	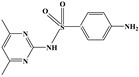	279→203 ^a^, 217.8 ^a^ 279→203 ^b^	
Thiabendazole	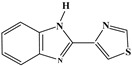	202→175 ^a^, 131 ^a^ 202→175 ^b^	
Amantadine	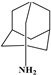	152.2→135 ^a^, 107 ^a^ 152.2→135 ^b^	
Ractopamine	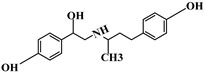	302→164 ^a^, 284 ^a^ 302→164 ^b^	
Clenbuterol	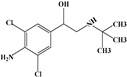	277→203 ^a^, 259 ^a^ 277→203 ^b^	
Bisacodyl	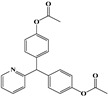	362→226 ^a^, 184 ^a^ 362→226 ^b^	
Metronidazole	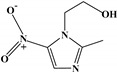	172→81.8 ^a^, 127.7 ^a^ 172→81.8 ^b^	

(^a^ qualitative ion, ^b^ quantitative ion).

**Table 2:** The standard calibration curves, linear range, limit of detection (LOD), limit of quantitation (LOQ), and repeatability of the SBA-15-SPME Arrow–PMS method for the determination of five veterinary drugs in milk and chicken.

	Analytes	Calibration Curves	Linear Range (μg kg^−1^)	R	LOD (μg kg^−1^)	LOQ (μg kg^−1^)	Repeatability (RSD, %)	
							Intra-Day (n = 3)	Inter-Day (n = 3)
Milk	Amantadine	y = 12.55x + 24.64	10–1000	0.9955	2.8	10	10.3	10.4
	Thiabendazole	y = 4.48x + 16.74	28–1000	0.9965	9.3	28	3.2	10.4
	Clenbuterol	y = 30.14x + 56.87	10–1000	0.9957	3	10	4.3	8.5
	Sulfamethazine	y = 116.7x + 62.51	10–1000	0.9979	2.8	10	5.8	12.1
	Ractopamine	y = 19.54x + 8.53	10–1000	0.9951	2.8	10	7.5	11.8
Chicken	Amantadine	y = 9.47x + 13.45	12–1000	0.9954	3.6	12	8.7	13.4
	Thiabendazole	y = 3.93x + 31.51	35–1000	0.9966	11.7	35	8.2	9.2
	Clenbuterol	y = 1.82x + 16.19	13–1000	0.9976	4.3	13	7.4	9.1
	Sulfamethazine	y = 92.19x + 39.89	12–1000	0.9989	3.6	12	4.7	11.0
	Ractopamine	y = 16.33x + 0.78	12–1000	0.9981	3.5	12	7.3	9.4

**Table 3:** Analytical results for the determination of five veterinary drugs in milk and chicken samples.

Analytes	Maximum Residue Limits				Milk			Chicken		
					Spiked Concentration (μg kg^−1^)			Spiked Concentration (μg kg^−1^)		
	China	European Union	United States		25	100	200	25	100	200
Amantadine	0 µg kg^−1^	0 µg kg^−1^	0 µg kg^−1^	Recovery (%)	87	102	102	99	88	102
				RSD (n = 3)	13.4	5.4	6.3	8.3	13.9	4.6
Clenbuterol	0 µg kg^−1^	0 µg kg^−1^	0 µg kg^−1^	Recovery (%)	92	100	99	105	103	85
				RSD (n = 3)	5.5	7.6	2.6	7.5	3.8	4.5
Sulfamethazine	100 µg kg^−1^ in muscle, 25µg kg^−1^ in milk, and 10 µg kg^−1^ in egg	100 µg kg^−1^ in muscle	100 µg kg^−1^ in muscle	Recovery (%)	97	107	98	111	98	85
				RSD (n = 3)	14.7	5.3	10.3	3.2	6.4	2.2
Ractopamine	0 µg kg^−1^	0 µg kg^−1^	50 µg kg^−1^ in muscle and 10 µg kg^−1^ in liver	Recovery (%)	95	106	100	116	106	98
				RSD (n = 3)	12.4	11.2	7.0	10.1	12.5	2.7
					Milk			Chicken		
					Spiked concentration (μg kg^−1^)			Spiked concentration (μg kg^−1^)		
					30	100	300	30	100	300
Thiabendazole	100 µg kg^−1^ in muscle, liver, and milk	100 µg kg^−1^ in muscle and milk	50 µg kg^−1^ in muscle and 100 µg kg^−1^ in liver	Recovery (%)	101	95	102	113	105	97
				RSD (n = 3)	8.3	3.4	8.7	2.1	4.6	4.3

**Table 4:** Comparison of the reported methods and the method developed in this study.

Methods	Sample	LODs (μg kg^−1^)	LOQs (μg kg^−1^)	Linear Range (μg kg^−1^)	Analytes	Recovery (%)	Detection Time (min)	References
^a^ UA-LLME-DES-HPLC-UV	Animal feed	20–50	100	100–50,000	SAs	88.1–97.8	23.5	[44]
^b^ RP-HPLC-UV-IAC	Chicken and egg	14.1–45	46.9–150	150–10,000	SAs	78.2–105.2	46	[45]
^c^ LC-MS/MS	Fruit juices	–	1.0	0.5–20	AMD	79.9–91.5	15	[46]
^d^ EME-LC-MS/MS	Animal derived foods	0.07–0.11	0.23–0.36	1–1000	RAC	80.3–108.8	10	[47]
SPME-PMS	Milk Chicken	2.8–9.3 3.5–11.7	10–28 12–35	10–1000 12–1000	SM2, RAC, TBZ, CL, AMD	85–116	0.5	This work

^a^ Ultrasound assisted liquid–liquid microextraction in combination with hydrophobic deep eutectic solvent high-performance liquid chromatography UV detection. ^b^ Reversed-phase high-performance liquid chromatography immunoaffinity chromatography. ^c^ Liquid chromatography—tandem mass spectrometry. ^d^ Electromembrane extraction and liquid chromatography tandem mass spectrometry. Sulfonamides (SAs), amantadine (AMD), ractopamine (RAC), clenbuterol (CL), thiabendazole (TBZ), and sulfamethazine (SM2).

## Data Availability

The original contributions presented in the study are included in the article/Supplementary Material, further inquiries can be directed to the corresponding author.

## Supplementary Materials

The following supporting information can be downloaded at: https://www.mdpi.com/article/10.3390/foods13203337/s1, text S1. Synthesis of SBA-16, MCM-41, and UIO-66 series materials; Figure S1: Optimization of SPME Arrow coating procedures: (a) material proportion in the electrospinning solution, (1) 0.1 g PAN + 0.1 g SBA-15 + 2 mL DMF, (2) 0.1 g PAN + 0.2 g SBA-15 + 3 mL DMF, (3) 0.1 g PAN + 0.3 g SBA-15 + 4 mL DMF, (4) 0.2 g PAN + 0.1 g SBA-15 + 3 mL DMF, (5) 0.2 g PAN + 0.2 g SBA-15 + 4 mL DMF, (6) 0.3 g PAN + 0.1 g SBA-15 + 4 mL DMF; (b) electrospinning time; Figure S2. SEM images of SBA-15-SPME Arrow at different spinning times (a) Bare SPME Arrow (diameter = 0.98 mm); (b) 20 min (coating thickness = 0.27 mm); (c) 30 min (coating thickness = 0.34 mm); (d) 50 min (coating thickness = 0.6 mm); (e) 60 min (coating thickness = 0.53 mm); Figure S3. Optimization of the extraction and desorption conditions of PDMS Smart SPME Arrow: (a) extraction time, (b) desorption solvents (1. methanol:water = 7:3, 2. acetonitrile:water = 7:3, 3. methanol:water = 9:1, 4. acetone:water = 7:3) and (c) desorption time; Figure S4. Optimization of the extraction and desorption conditions of Polyacrylate Smart SPME Arrow: (a) extraction time, (b) desorption solvents (1. methanol:water = 7:3, 2. acetonitrile:water = 7:3, 3. methanol:water = 9:1, 4. acetone:water = 7:3) and (c) desorption time; Figure S5. Optimization of the extraction and desorption conditions of Carbon WR/PDMS Smart SPME Arrow: (a) extraction time, (b) desorption solvents (1. methanol:water = 7:3, 2. acetonitrile:water = 7:3, 3. methanol:water = 9:1, 4. acetone:water = 7:3) and (c) desorption time; Figure S6. Optimization of the extraction and desorption conditions of DVB/Carbon WR/PDMS Smart SPME Arrow: (a) extraction time, (b) desorption solvents (1. methanol:water = 7:3, 2. acetonitrile:water = 7:3, 3. methanol:water = 9:1, 4. acetone:water = 7:3) and (c) desorption time; Figure S7 Optimization of the extraction and desorption conditions of DVB/PDMS Smart SPME Arrow: (a) extraction time, (b) desorption solvents (1. methanol:water = 7:3, 2. acetonitrile:water = 7:3, 3. methanol:water = 9:1, 4. acetone:water = 7:3) and (c) desorption time; Figure S8. Carryover test of the SBA-15-SPME Arrow with five veterinary drugs.
