# Supplementary material for: Detection of Veterinary Drugs in Food Using a Portable Mass Spectrometer Coupled with Solid-Phase Microextraction Arrow
Source: Foods. 2024 Oct 21;13(20):3337. doi: 10.3390/foods13203337 (PMC11507621; doi:10.3390/foods13203337)
Supplement: Supplementary file 1 [file foods-13-03337-s001.zip › foods-3245072-supplementary.pdf]

Supplementary Material

Detection of Veterinary Drugs in Food Using a Portable Mass Spectrometer Coupled with Solid-Phase Microextraction Arrow

Hangzhen Lan [1,*], Xueying Li [1], Zhen Wu [1], Daodong Pan [1], Ning Gan [2] and Luhong Wen [3]

[1] State Key Laboratory for Managing Biotic and Chemical Threats to the Quality and Safety of Agro-Products, Zhejiang Key Laboratory of Intelligent Food Logistic and Processing, Zhejiang-Malaysia Joint Research Laboratory for Agricultural Product Processing and Nutrition, College of Food Science and Engineering, Ningbo University, Ningbo 315800, China; lixueying@nbu.edu.cn (X.L.); wuzhen@nbu.edu.cn (Z.W.); pandaodong@nbu.edu.cn (D.P.)

[2] School of Material Science and Chemical Engineering, Ningbo University, Ningbo 315211, China; ganning@nbu.edu.cn

[3] China Innovation Instrument Co., Ltd., Ningbo 315000, China; wenluhong@nbu.edu.cn

* Correspondence: lanhangzhen@nbu.edu.cn

S1. Synthesis of SBA-16, MCM-41, and UIO-66 series materials

1.25 g CTAB was dissolved in 490 mL of 14 wt% NH_4OH and stirred for 5 min to make it fully dissolved. Then 10 mL of TEOS was slowly added into the solution, and a white precipitate gradually appeared, and stirred at room temperature for 2 h. Then the precipitate was filtered, dried, and finally roasted in air at 550 °C for 6 h to remove the template to obtain MCM-41.

6.66 g of F127 was taken in a three-necked flask, 200 mL of 2 mol L^{-1} hydrochloric acid solution was added, and the solution was stirred thoroughly in a water bath at 35 °C to dissolve the F127 completely, after which 25 mL of TEOS was added dropwise. After that, 25 mL of TEOS was added drop by drop, and the mixture was stirred in a water bath at 35 °C for 20 h. The white dispersion was poured into a polytetrafluoroethylene container, which was placed in a high-pressure reactor, placed in a muffle furnace, and reacted by hydrothermal synthesis at 100 °C for 24 h. After completion of the reaction, the solution was filtered, and the white precipitate was obtained, which was cleaned with deionized water, and the cleaned material was dried at 70 °C. The cleaned material was dried at 70 °C overnight. The dried material was placed in a crucible and calcined in a muffle furnace at 550 °C for 6 h to remove the template to obtain SBA-16.

UiO-66-NH_2-50% was synthesized by dispersing zirconium chloride (320 mg), 2-aminoterephthalic acid (125 mg), terephthalic acid (125 mg), acetic acid (9.864 g) and HCl (270 mg) in DMF (50 mL). Subsequently, the mixture was reacted at 120 °C for 24 h. The solid product was washed five times with DMF and ethanol, respectively. Finally, UiO-66-NH_2-50% was vacuum dried at 150 °C for 12 h. The sample containing 100 mg of UiO-66-NH_2-50% was heat-treated at 350 °C for 2 h. After cooling to room temperature, the new material was vacuum dried at 150 °C for 12 h to obtain Meso-UiO-66. Meso-UiO-66 (100 mg) and HCl (0.2 mL) were mixed into DMF (20 mL), which was heated at 50 °C and stirred for 12 h. The solid product was washed by DMF and ethanol for five times, respectively. After centrifugation, the new material was dried under vacuum at 150 °C for 12 h and named HCl-meso-UiO-66; 45 mg of HCl-meso-UiO-66, 60 mg of 4-pentylbenzoic acid and 3 mL of DMF were

added to a glass vial, capped, and stirred at 80 °C for 24 h. The solid product was washed by DMF and ethanol for 5 times. The solid product was washed five times by DMF and ethanol, respectively, and after centrifugation, the new material was dried under vacuum at 150 °C for 12 h and named 4-meso-UiO-66.

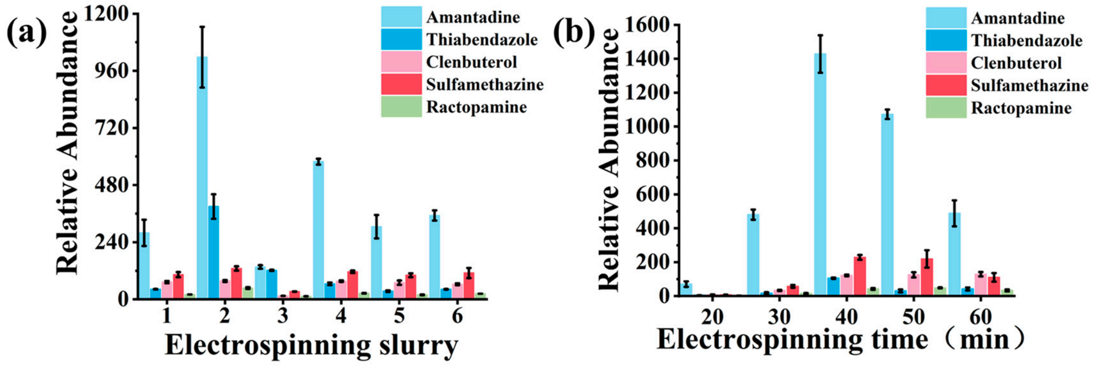

Figure S1. Optimization of SPME Arrow coating procedures: (a) material proportion in the electrospinning solution, (1) 0.1 g PAN+0.1 g SBA-15+2 mL DMF, (2) 0.1 g PAN+0.2 g SBA-15+3 mL DMF, (3) 0.1 g PAN+0.3 g SBA-15+4 mL DMF, (4) 0.2 g PAN+0.1 g SBA-15+3 mL DMF, (5) 0.2 g PAN+0.2 g SBA-15+4 mL DMF, (6) 0.3 g PAN+0.1 g SBA-15+4 mL DMF; (b) electrospinning time.

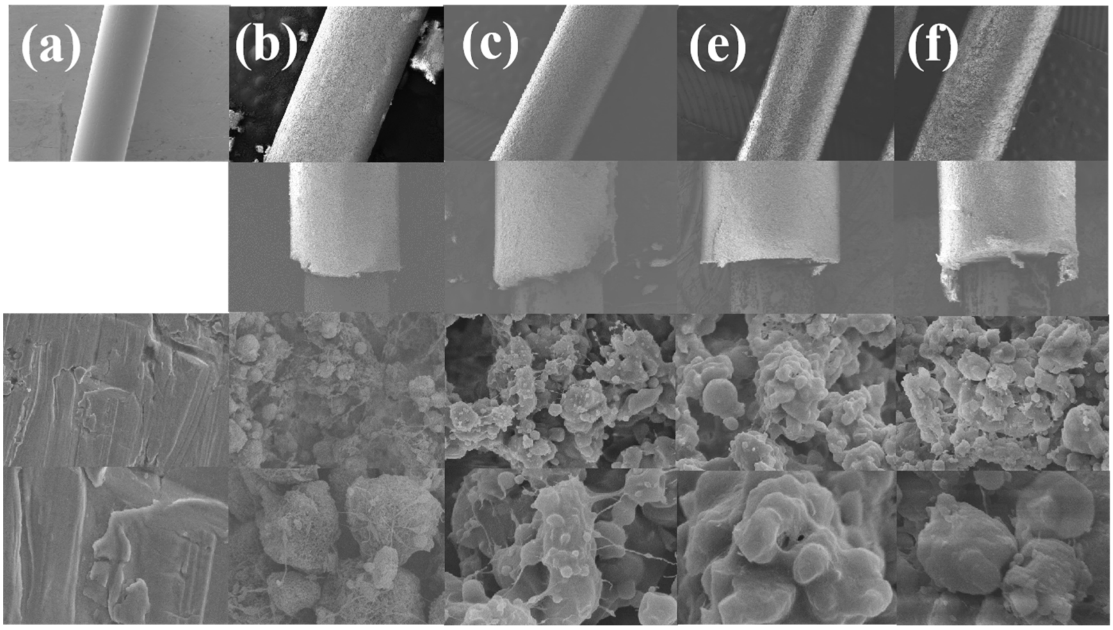

Figure S2. SEM images of SBA-15-SPME Arrow at different spinning times (a) Bare SPME Arrow (diameter=0.98 mm); (b) 20 min (coating thickness=0.27 mm); (c) 30 min (coating thickness=0.34 mm); (d) 50 min (coating thickness=0.6 mm); (e) 60 min (coating thickness=0.53 mm).

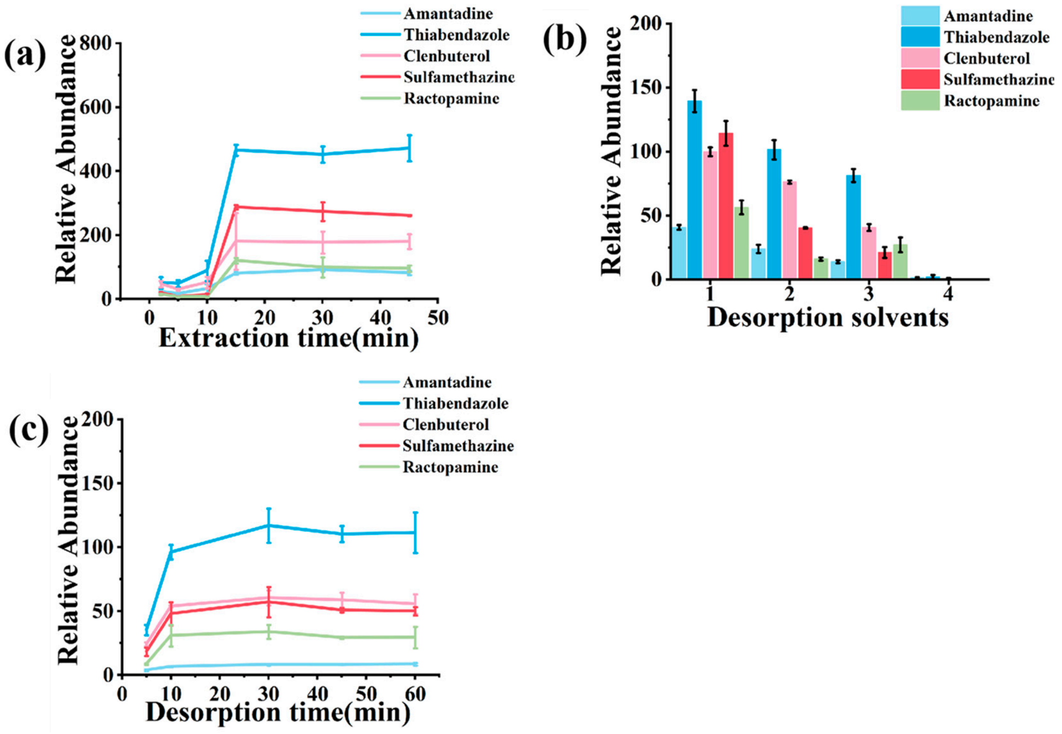

Figure S3. Optimization of the extraction and desorption conditions of PDMS Smart SPME Arrow: (a) extraction time, (b) desorption solvents (1. methanol:water=7:3, 2. acetonitrile:water=7:3, 3. methanol:water=9:1, 4. acetone:water=7:3) and (c) desorption time.

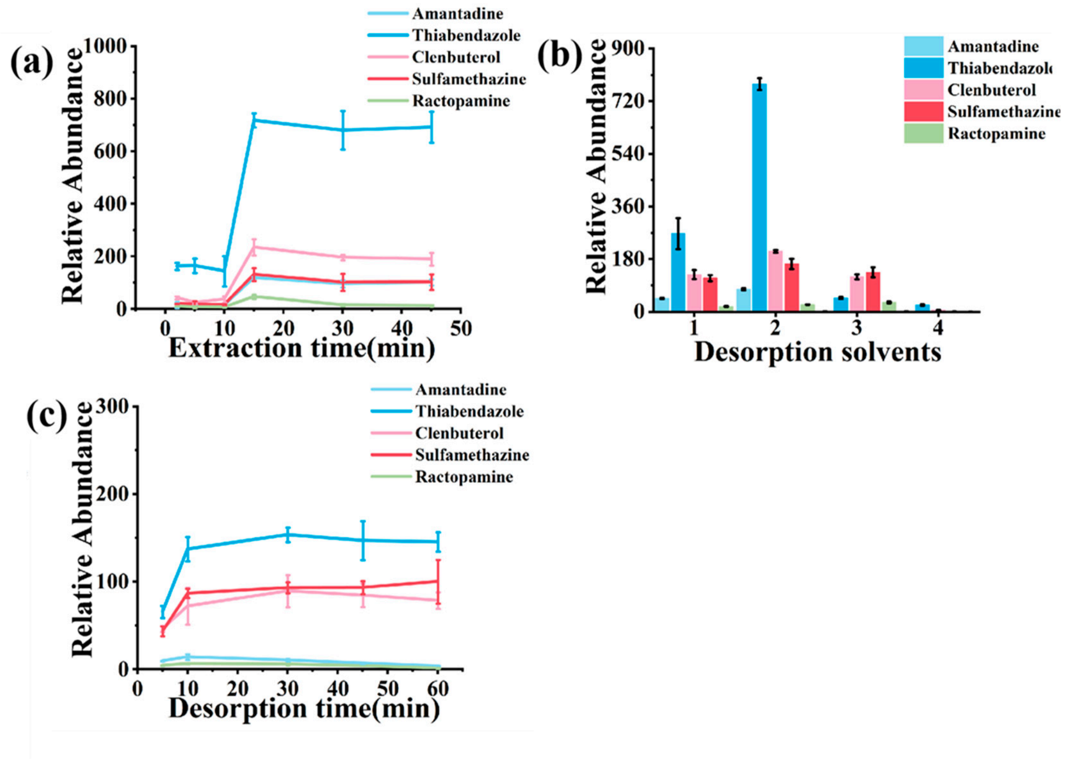

Figure S4. Optimization of the extraction and desorption conditions of Polyacrylate Smart SPME Arrow: (a) extraction time, (b) desorption solvents (1. methanol:water=7:3, 2. acetonitrile:water=7:3, 3. methanol:water=9:1, 4. acetone:water=7:3) and (c) desorption time.

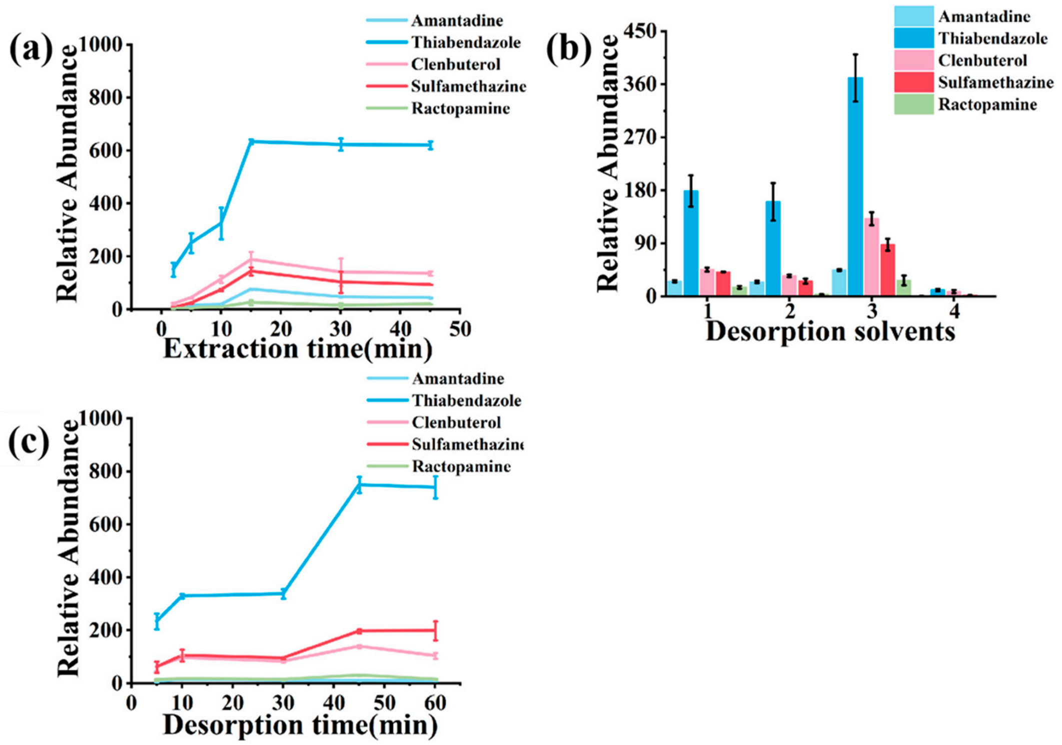

Figure S5. Optimization of the extraction and desorption conditions of Carbon WR/PDMS Smart SPME Arrow: (a) extraction time, (b) desorption solvents (1. methanol:water=7:3, 2. acetonitrile:water=7:3, 3. methanol:water=9:1, 4. acetone:water=7:3) and (c) desorption time.

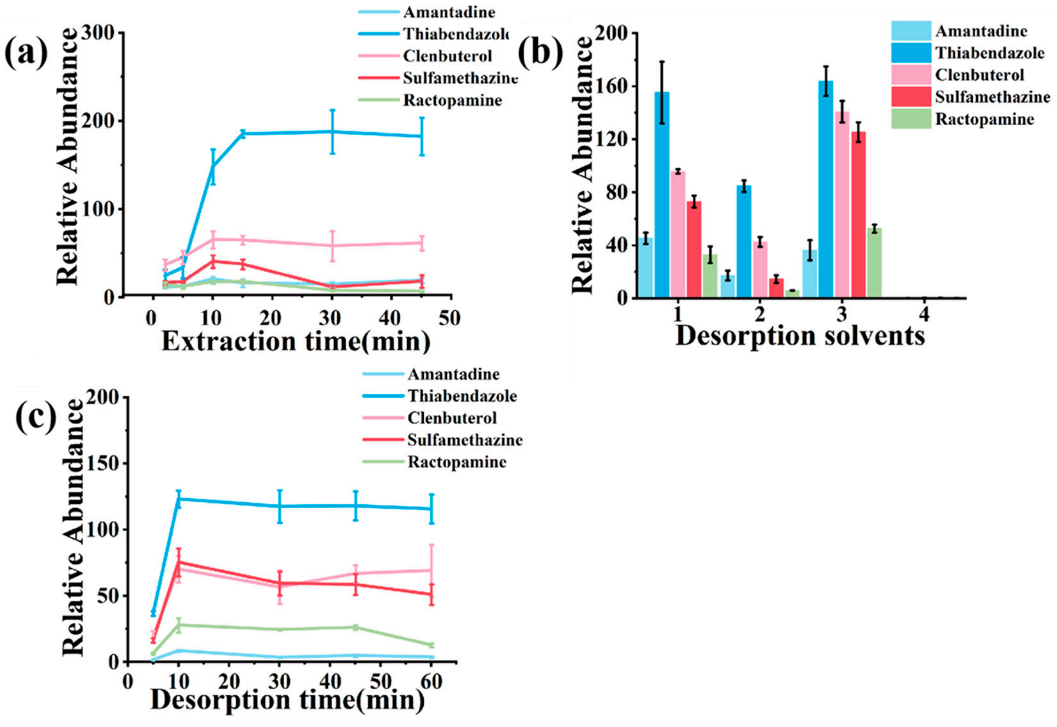

Figure S6. Optimization of the extraction and desorption conditions of DVB/Carbon WR/PDMS Smart SPME Arrow: (a) extraction time, (b) desorption solvents (1. methanol:water=7:3, 2. acetonitrile:water=7:3, 3. methanol:water=9:1, 4. acetone:water=7:3) and (c) desorption time.

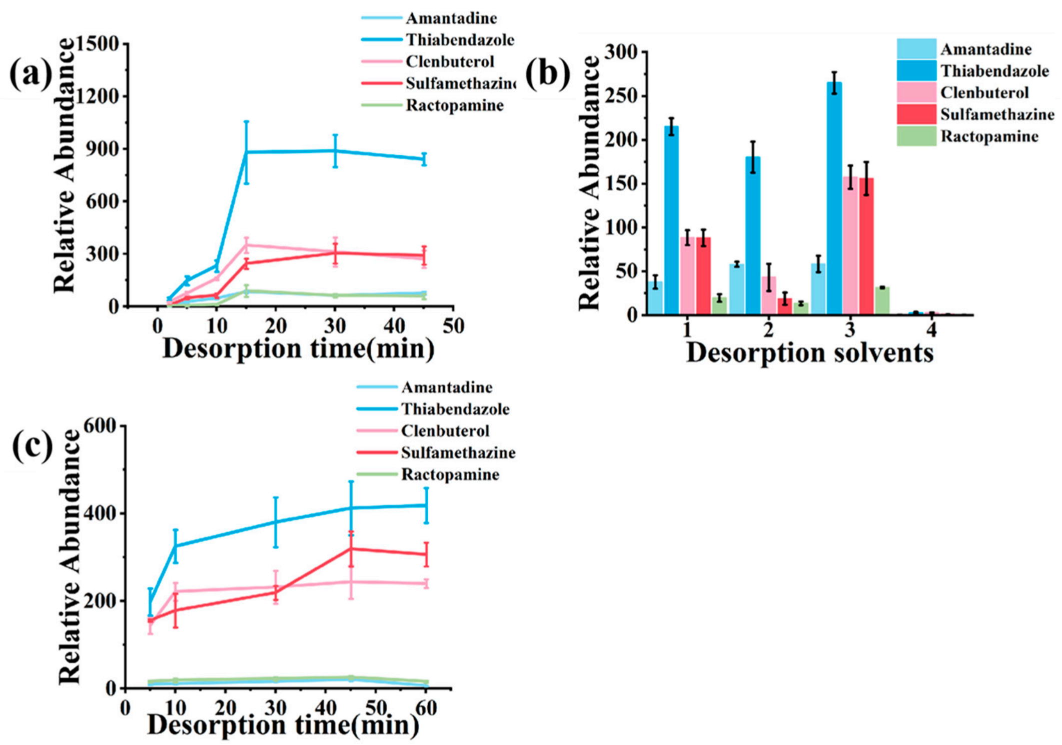

Figure S7 Optimization of the extraction and desorption conditions of DVB/PDMS Smart SPME Arrow: (a) extraction time, (b) desorption solvents (1. methanol:water=7:3, 2. acetonitrile:water=7:3, 3. methanol:water=9:1, 4. acetone:water=7:3) and (c) desorption time.

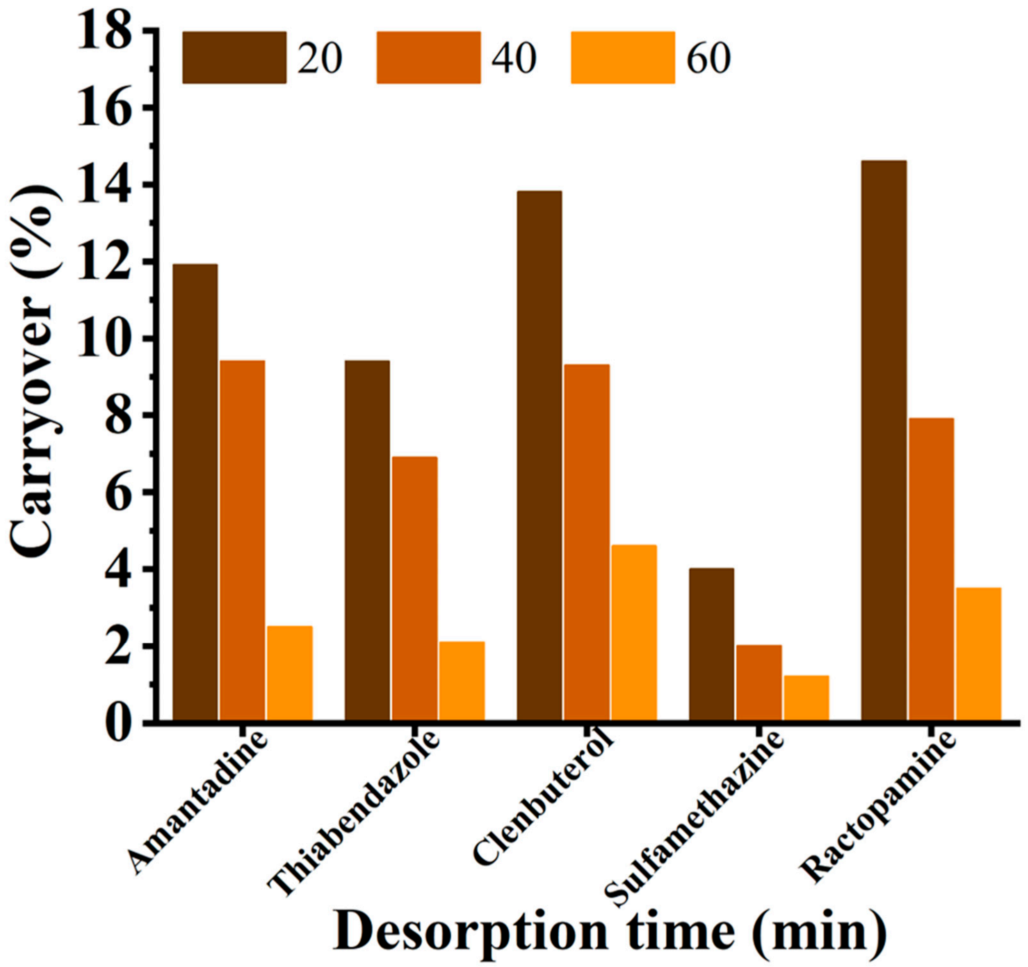

Figure S8. Carryover test of the SBA-15-SPME Arrow with five veterinary drugs.
